# Does Urinary Catheter Replacement Prior To Obtaining Urine for Culture Make a Difference?

**DOI:** 10.1017/ash.2024.195

**Published:** 2024-09-16

**Authors:** Julia Moody, Kenneth Sands, Keetha Kratzer, Eunice Blanchard, Laura McLean

**Affiliations:** HCA Healthcare; Hospital Corporation of America; HCA Healthcare Corporate Office

## Abstract

**Background:** Indwelling urinary catheters (UCs) generate biofilm that grows over time, raising concern that after several days any culture from a UC may generate a false positive result. Whether and when to replace a UC prior to culture is controversial, with prior studies recommending anywhere from 3 to 14 days as appropriate, but with no conclusive data. We evaluated urine culture results across a large healthcare system where, beginning in 2019, some facilities adopted the practice of changing UC before collection if indwelling for 3 days or more. **Method:** Analysis was from nursing electronic health record documentation of UC changes and urine cultures collected on patients with indwelling UC in 2022. UC changes were defined as a stop followed by a start within 12 hours. Patient exclusion criteria included a UC other than “temporary/indwelling” and surgical procedure during the admission. Statistics applied Pearson’s Chi-squared test with Yates continuity correction using R Core Team (2023) R: A Language and Environment for Statistical Computing. **Result:** Total UC episodes meeting criteria was 88,347 across 152 acute care hospitals. Episodes in days was 0-3 for 65%, 4-9 for 29% and >9 for 6%. Most urine cultures were taken at 3 days (p UC Changed? Culture Negative Culture Positive No 4916 (98.8%) 61 (1.2%) Yes 588 (98.7%) 8 (1.3%) Cultures were positive at the same rate whether a UC change occurred or not at >3 days (p=0.96). No difference was found in NHSN reported CAUTI prevalence among the UC change vs. no change in the >3 day groups. **Conclusion:** Urine culture results do not appear to be impacted by UC change as early as 3 days. UC change without benefit may generate unnecessary costs and complications.

